# Normokalemic Thyrotoxic Periodic Paralysis with Acute Resolution in the Emergency Department

**DOI:** 10.5811/cpcem.2017.1.33211

**Published:** 2017-03-15

**Authors:** James I. Gragg, Massimo Federico, Larry B. Mellick

**Affiliations:** * Carl R. Darnall Army Medical Center, Department of Emergency Medicine, Fort Hood, Texas; † Augusta University Medical Center, Department of Emergency Medicine, Augusta, Georgia

## Abstract

Thyrotoxic periodic paralysis is a rare cause of acute paralysis in the emergency department (ED). The disorder is generally thought to be due to acute hypokalemia leading to paralysis. Treatment is generally targeted at correcting the thyrotoxic state with careful potassium repletion. We present a rare case of normokalemic, thyrotoxic periodic paralysis with acute resolution while in the ED.

## INTRODUCTION

Thyrotoxic periodic paralysis (TPP) is a lesser known cause of intermittent, potentially curable, muscle weakness. The presentation is most commonly seen in Asian men and is usually associated with hypokalemia, which is believed to play a role in the underlying pathophysiology. The diagnosis is usually not made in the emergency department (ED). Instead, it is made after hospital admission when electrolyte abnormalities are corrected, anti-thyroid treatments are initiated and the weakness improves. Although extremely rare, fatalities have been reported. These fatalities may occur through two possible mechanisms: weakness significantly affecting the respiratory muscles leading to respiratory arrest, or severe hypokalemia leading to ventricular tachycardia or fibrillation.^1^ Patients must be monitored closely for evidence of respiratory compromise or the development of life-threatening arrhythmias. Clinicians should include this disease process in their differential diagnoses along with other paralyzing disorders such as Guillain-Barré, multiple sclerosis, transverse myelitis, tick paralysis, conversion disorder or hysteria.

Treatment of TPP is not well established and must be individualized to each presentation. Even though potassium supplementation has traditionally been used to hasten recovery of weakness, caution is advised because significant rebound hyperkalemia may result. Treatment of the underlying thyroid disorder should also be addressed. In this paper we present a case of TPP in a patient with a normal potassium level who recovered fully while in the ED.

## CASE REPORT

A 26-year-old black, male, active duty soldier presented to the ED via emergency medical services for evaluation of bilateral lower extremity weakness. The patient reported that his symptoms began earlier in the morning while he was running with his unit during physical fitness training. Throughout the course of the three-mile run he described being unable to maintain pace and felt weighed down by his legs. At the completion of the training his symptoms worsened and when he attempted to get out of his car, he could not move his legs. Specifically, he reported that he could not move either of his thighs or lower extremities, but maintained motion of his feet and toes. Unable to move, bystanders assisted him to his local clinic where he was assessed and then emergently brought to the ED for continued evaluation.

Upon initial assessment the patient had a mild tachycardia but with otherwise normal vital signs. Neurological assessment demonstrated intact cranial nerves, absence of cerebellar signs, and preserved upper extremity motor and sensory exams. On examination of his lower extremities, he had 2/5 strength of hip flexion, hip extension, and knee extension. Dorsi- and plantar-flexion of the feet were intact bilaterally. Sensory exam was intact to light touch throughout his lower extremities. Gait was unable to be assessed secondary to his inability to ambulate or stand without assistance.

His past medical history was significant only for a dermatitis that was treated with triamcinolone and over-the-counter medications. He did admit to using multiple supplements two to three times daily to boost muscle strength, but took no prescribed daily medications. He denied any other symptoms, recent illnesses, vaccinations, travel, military deployments or prior neurologic symptoms. Family history was significant for systemic lupus erythematosus and an unknown thyroid disorder in his mother, as well as an unknown thyroid disorder in his younger brother. Laboratory investigations were significant for a urine specific gravity of 1.032, a thyroid stimulating hormone level below assay detection, free thyroxine level of 18.8 ng/dL (0.7–1.9 ng/dl), creatine kinase of 501 U/L, aspartate aminotransferase of 55 U/L, alanine aminotransferase of 71 U/L, and phosphate level of 2.2 mg/dL. Importantly, his potassium level was normal at a level of 3.8 mmol/L. Electrocardiogram was significant for sinus tachycardia and left ventricular hypertrophy. After one liter of normal saline, the patient fully regained muscle strength, including the abilities to ambulate without difficulty, perform squats, single-leg stance and jumping jacks.

Given complete resolution of symptoms and abnormal thyroid function tests, the patient was discharged with a diagnosis of normokalemic, thyrotoxic periodic paralysis. In consultation, he was initiated on propranolol 10 mg three times daily and methimazole 20 mg daily. Outpatient follow-up with endocrinology was also secured, with subsequent testing demonstrating elevations in free triiodothyronine (FT3) at 12.2 pg/mL (1.7–3.7 pg/mL), thyroperoxidase antibody at 209 IU/mL (0–34 IU/mL), and percent thyroid-stimulating immunoglobulins of 294% (0–139%). A diagnosis of Graves’ disease was made and at follow-up the patient continued to do well. Five months after his initial presentation, he has required only minor adjustments in medication, without any repeated neurologic deficits.

## DISCUSSION

This case represents a unique presentation of TTP, as well as a unique resolution of symptoms while in the ED. The disease process has been well described in the Asian population, where among patients with thyrotoxicosis there is an incidence of roughly 2%. TTP is now more commonly recognized in non-Asian populations; however, the incidence is still only 0.1% of patients with thyrotoxicosis.^2^ Heavy carbohydrate loads or extreme exercise are thought to play a role in the thyrotoxic-primed pathophysiology of the disorder. With hyperthyroidism the Na^+^/K^+^ ATPase is upregulated in skeletal muscle, which serves as a large potassium reservoir in the body. In the setting of a large carbohydrate load the resulting hyperinsulinemia further activates the Na^+^/K^+^ ATPase, thereby driving potassium into the cell. Similarly, with exercise, endogenous catecholamines and adrenergic stimulation activate the Na^+^/K^+^ ATPase. Both scenarios lead to a sequestration of potassium in skeletal muscle that results in the relative hypokalemia and paralysis observed in these patients.^3^ Our patient, however, had a potassium level within the normal range, thus calling into question the requirement of hypokalemia in making the diagnosis. However, significant shifts of potassium most likely still take place at the muscle cellular level. And, another case report demonstrated that normokalemia can be present on admission. However, in that case potassium levels continued to fall during admission.^4^ Conversely, our patient’s potassium remained normal on repeat laboratory tests. Normokalemia, therefore, should not be used as a single laboratory evaluation to rule out TTP.

The treatment of TTP is not well defined. Traditionally, potassium repletion has been the mainstay of treatment. Studies suggest that restoration of potassium levels leads to resolution of paralysis, with potassium decreasing the time to resolution from 13 hours to six hours.^5^ However, the physiology of TTP suggests that patients are not truly deficient in potassium stores and exogenous potassium repletion can cause rebound hyperkalemia. Clinicians choosing to give potassium should be prepared to monitor for and treat hyperkalemia. However, since the potassium level may actually continue to fall despite potassium supplementation, careful monitoring is in order as heart block and cardiogenic shock from hypokalemia may result.^6^ The typical course of the disease process is still not well defined and continued research is needed. For now, it is best to closely monitor these patients and frequently repeat laboratory tests to determine electrolyte trends. Finally, in conjunction with hypokalemia, a concurrent hypomagnesemia or hypophosphatemia may also be present.^7^

Treatment should also address the underlying thyrotoxic state. As in thyroid storm, given its non-selective pharmacology, propranolol is the preferred beta-blocker. Blockade of the adrenergic hyperstimulation halts the Na^+^/K^+^ ATPase, preventing further potassium sequestration. Propranolol alone has been shown to effectively reverse both the paralysis and electrolyte abnormalities in TTP.^8^ Given the inherent risks of large-dose potassium repletion in the absence of severe hypokalemia, it may be reasonable to withhold supplementation while treating with propranolol. Evaluation of the etiology of the thyrotoxic state should also be undertaken with treatments targeting the underlying cause. Graves’ disease remains the most common precipitating cause, as was seen in our patient.

## CONCLUSION

Thyrotoxic periodic paralysis is a lesser known disease that should be considered in any patient with an unexplained, objective weakness. While hypokalemia is commonly reported, it should be recognized that normokalemia may also occur. Treatment is aimed at correcting the underlying thyrotoxic state and low serum levels of potassium. However, potassium repletion must be undertaken with extreme caution to avoid rebound hyperkalemia and arrhythmia. Finally, even though the condition is most commonly seen in Asian men, TTP may also be found in non-Asian populations as occurred with our patient.
